# Effect of Imiquimod on Tachyzoites of *Toxoplasma gondii* and Infected Macrophages *in vitro* and in BALB/c Mice

**DOI:** 10.3389/fcimb.2020.00387

**Published:** 2020-07-31

**Authors:** Leila Zaki, Fatemeh Ghaffarifar, Zohreh Sharifi, John Horton, Javid Sadraei

**Affiliations:** ^1^Department of Parasitology, Faculty of Medical Sciences, Tarbiat Modares University, Tehran, Iran; ^2^Blood Transfusion Research Center, High Institute for Research and Education in Transfusion Medicine, Tehran, Iran; ^3^Tropical Projects, Hitchin, United Kingdom

**Keywords:** *Toxoplasma gondii*, imiquimod, macrophages, *in vivo*, *in vitro*

## Abstract

Treatment for toxoplasmosis is not completely successful because of their unwanted side effects, and new treatments are needed. Imiquimod has ability to moderate immune response and used to treat a wide variety of infections and tumors. The aim of the present study was to evaluate the effect of imiquimod on the tachyzoites of *T. gondii* and infected macrophages *in vitro* and in BALB/c mice. The viability of *T. gondii* was assessed in the presence of various concentrations of imiquimod by direct counting after 6 and 24 h. The MTT assay was used to identify the viability of uninfected macrophages. The apoptotic effects were determined with flow cytometry on the tachyzoites and infected macrophages. For evaluation of parasite load in pre-treatment or post-treatment of macrophages Quantitative real time PCR (qPCR) was performed. For *in vivo* experiments, BALB/c mice received imiquimod before and after challenge with parasites. The mortality rate of mice, parasite numbers in spleen, and the INF-γ and IL-4 cytokine levels in spleen lymphocytes were evaluated. Imiquimod demonstrated anti-*Toxoplasma* effects by reducing the number of tachyzoites. The results of flow cytometry for drug-treated tachyzoites showed that apoptosis did not rise significantly relative to the control group (*p* < 0.05). Moreover, apoptosis was enhanced in infected macrophages as the concentration of imiquimod was reduced. The parasitic burden in imiquimod pretreated macrophages was significantly lower than those treated after infection (*p* < 0.01). A marked reduction was observed in survival rate, parasite load and INF-γ level in BALB/c mice that received imiquimod before parasitic challenge relative to those received drug after parasitic challenge (*p* < 0.01). Overall, imiquimod in the pretreated group had greater anti-*Toxoplasma* effects than imiquimod in posttreated group *in vitro* and *in vivo*. imiquimod may be considered as a candidate for use against Toxoplasmosis both therapeutically and prophylactically.

## Introduction

*Toxoplasma gondii*, a ubiquitous apicomplexan parasite, causes a neglected parasitic disease known as toxoplasmosis which affects a significant portion of the human and animal population worldwide (Dubey et al., 2020). It is a major public health concern and it is estimated that more than 30 percent of people in the world are infected with *T. gondii*.

The infection can be contracted in a number of different ways, including consumption of undercooked meat containing tissue cysts, ingestion of contaminated water or food containing mature oocytes, and congenitally by transfer from a pregnant women to her fetus (Mendez and Koshy, 2017; Fallahi et al., 2018). It may also occur in individuals who received blood transfusions or organ transplantation from infected individuals.

In humans, toxoplasmosis is usually causes asymptomatic in immunocompetent persons, although it can lead to mild clinical manifestations such as swollen lymph nodes and flu-like symptoms (Gharavi et al., 2011; Gigley, 2016). In those with a weakened immune system (patients with malignancies, those with HIV/AIDS and organ transplant recipients), toxoplasmosis can cause widespread signs of differing severity such as encephalitis, seizures, vision disorders, poor coordination or even can contribute to the cause of death if untreated (Gigley, 2016). In addition, in pregnant mothers that acquire the infection during pregnancy, toxoplasmosis may cause miscarriage or neuropsychological manifestations, including hydrocephalus, blindness, mental retardation, encephalitis, etc. in newborns with congenital infection (Fishman, 2013; Wang et al., 2017).

Few effective control strategies are available for prevention and control of toxoplasmosis and unfortunately treatments are not completely satisfactory. The recommended treatment for toxoplasmosis is a combination of pyrimethamine (PYR) and sulfadiazine (SDZ) which is associated with unwanted side effects including significant toxicity, prolonged treatment, high cost and potential parasite resistance. Alternative agents such as clarithromycin, atovaquone, azithromycin, dapsone, co-trimoxazole (trimethoprim-sulfamethoxazole) are also used. However, treatment is unable to eradicate bradyzoites in tissue cysts (Montazeri et al., 2016; Montoya and Gomez, 2016; Foroutan et al., 2019). Therefore, it is of great importance for researchers to identify novel anti-*Toxoplasma* drugs with specific activity that could eliminate both tachyzoites and tissue cysts.

Although humoral immune plays a role against *T. gondii* infection, cellular immunity plays a crucial role in controlling and restricting growth of the parasite in both acute and chronic infection stages. Protection against toxoplasmosis generally is developed through both CD4+ and CD8 + T cells and production of cytokines from immune system cells (Sasai et al., 2018).

As a member of non-nucleoside isocyclic imidazoquinoline amines, imiquimod has the ability to moderate immune response and therefore it is used to treat a wide variety of viral infections and tumors (Sidky et al., 1992). Although the mechanism of action of imiquimod is not well defined (Miller et al., 1999; Stanley, 2002), it can stimulate the immune system by helping to activate monocytes, macrophages and dendritic cells to produce chemokines and proinflammatory cytokines such as IL-12, TNF-α, IL-6, IL-1a and IFN-α (Buates and Matlashewski, 1999; Dockrell and Kinghorn, 2001; Khamesipour, 2014). It is possible that this compound also has a role in treating some autoimmune diseases in human such as Bechet's disease, multiple sclerosis and optic neuritis (Geisse et al., 2004; Jabari et al., 2019). The therapeutic effects of imiquimod on cutaneous leishmaniasis have been reported previously (Buates and Matlashewski, 1999, 2001; Mehravaran et al., 2020). No studies have been conducted to investigate the toxoplasmacidal activity of imiquimod. Accordingly, the current study was planned to evaluate the effect of imiquimod on *T. gondii* and infected macrophages *in vitro* and in BALB/c mice.

## Materials and Methods

### *In vitro* Assays

#### Parasite Culture and Harvesting

Tachyzoites of the virulent strain RH of *T. gondii* were obtained from the Parasitology Department of Tarbiat Modares University. Initially, tachyzoites of the virulent RH strain of *T. gondii* were maintained by serial passage in Vero cells in 50 cm^2^ flasks.

Tachyzoites were collected and washed three times with cold phosphate-buffered saline (PBS; pH 7.4) following centrifugation at 1,000 g for 10 min at 4°C. Parasite concentrations were determined by trypan blue exclusion in a hemocytometer (Neubauer) chamber and were used immediately in the experiments (Mikaeiloo et al., 2016).

#### Preparation of Drugs

Imiquimod powder was obtained from Toulouse France (Invivogen, Toulouse, France, Lot No: IMQ-37-01A) and 4 mg was dissolved in 1 ml of dimethyl sulfoxide (DMSO) (Daejung Korea, Lot No: D0096QJ5) as a stock solution and then 10, 1, 0.1, and 0.01 μg/ml dilutions were prepared with Dulbecco's Modified Eagle Medium (DMEM). Sulfadiazine and pyrimethamine (Sigma–Aldrich, St. Louis, USA) were diluted in DMSO and used as positive controls at concentrations of 40 and 1 μg/ml, respectively.

#### Cytotoxicity Evaluation on Tachyzoites

The anti-tachyzoite activity of imiquimod was evaluated by light microscopic examination to determine the optimum concentrations for further experiments., Tachyzoites (2 × 10^6^ cells/well, 100 μl) supplemented with complete RPMI 1640 medium in the presence of various concentrations (10, 1, 0.1, and 0.01 μg/ml) of imiquimod were seeded as triplicates in a 96-well microtiter plate. Parasites plus complete RPMI 1640 medium (without drug) were used as an untreated control while SDZ + PYR was used as the positive control. After 6 and 24 h incubation at 37°C, tachyzoites were directly counted in the hemocytometer chamber using a phase contrast microscope and the results from the experimental groups compared with those of the control groups.

#### Macrophage Culture

In the present work, the Raw.264.7 macrophage cell line, derived from mouse BALB/c monocyte macrophages, was obtained from Department of Medical Parasitology in Tarbiat Modares University of Tehran, Iran. These cells were first grown and propagated in cell culture flasks containing RPMI 1640 medium (Gibco, Germany) supplemented with 10% heat-inactivated FBS and 100 IU ml^−1^ of Penicillin and 100 μg ml^−1^ of streptomycin incubated at 37°C (5% CO_2_). Growth was observed every day by inverted microscope and when cells reached about 80–90% confluence, they were carefully trypsinized and sub-cultured into a new flask containing fresh culture medium.

#### Treatment and Collection of Infected Macrophage Cells

The study was conducted to investigate both therapeutic effects and preventive effects of imiquimod. For assessment of therapeutic effects, macrophages were seeded into 24-well microplates (1 ml per well with 1 × 10^5^ cells/well in 10% FCS RPMI 1640 medium) and incubated at a temperature of 37°C with 5% CO_2_ for 24 h. After adherence of the cells, they were infected using *T. gondii* in a ratio of 2:1 (parasites: macrophages) and the plates maintained under the previous conditions. Six hours after infection, the cells were washed twice with cold phosphate buffer saline (PBS) to remove unadhered macrophages from the wells and fresh culture medium was supplemented with the different concentrations of drugs. Next, the samples were collected and transferred in to a 1.5 mm DNase/RNase-free tubes and kept at a temperature of −70°C before estimating the parasite load.

To evaluate the preventive effects of imiquimod, the cultured macrophages were treated with the different concentrations of imiquimod before infection with tachyzoites. The remaining stages were accomplished as described above.We harvested the tachyzoites for pre and post treatment in the same condition.

#### Quantitative Real Time PCR (qPCR) for Assessment of Parasite Load in Infected Macrophages

For evaluation of parasite load in both the above *in vitro* models, RNA was extracted using the Qiagen RNA isolation kit (RNeasy Mini Kit, Qiagen). A nanodrop device (Roche-Germany) was used to determine the quantity and purity of the extracted RNA. Following this, cDNA was generated using a cDNA Synthesis Kit (Quanti Tect Reverse Transcription Kit, Qiagen) from RNA samples. To create the standard curve, samples of the real-time PCR assay, 6-fold-dilutions ranging from 2 × 10^1^ to 2 × 10^6^ parasites (total DNA extract from a sample containing 10^7^ tachyzoites of strain RH per ml) were prepared and then threshold cycle (Ct) values were calculated for these standard curves. SYBR-green real-time PCR using repetitive element (RE) gene primers were taken to calculate the number of parasites in the treated and control samples. Each sample was amplified in final volumes of twenty-five microliters containing 12.5 μl of SYBR Green PCR Master Mix (without ROX), 2 μl of template cDNA, 8.5 μl of injected distilled water, 1 μl of the forward primer (5′−AGG GAC AGA AGT CGA AGG GG−3′) and 1 μl of the reverse primer (5′−GCA GCC AAG CCG GAA ACA TC−3′) (Montazeri et al., 2016). Amplification was accomplished under the following conditions: 95°C for 15 min (initial step), 40 cycles at 95°C for 15 s (denaturation step), 60°C for 15 s (annealing step), and 72°C for 15 s (amplification step).

#### Toxicity of Imiquimod in Uninfected Macrophages

The MTT assay was used to determine the viability of macrophages exposed to imiquimod. Briefly, 1 mL of Raw.264.7 macrophage cells, suspended in RPMI 1640 medium enriched with 10 % FBS, were seeded at a concentration of 1 × 10^5^cells/well in 96-well microtiter plates and incubated in 5% CO_2_ and 95% humidity for 24 h. After adhering the cells, they were exposed to different concentrations of drug (from 10 to 0.01 μg/ml) and incubated for a further 24 h under the same conditions. After this step, 20 μL of solution of MTT (0.5 μg/ml) was added in to each well and incubated for 4 h. The plates were centrifuged for 10 min at 3,000 g and then the contents of each well was discharged slowly and replaced with 100 μL of dimethyl sulfoxide (DMSO) to distinguish between the viable cells (formazan formation) and decayed cells. Finally, absorbance at 570 nm of each well, using an ELISA reader, was evaluated within 30 min. The following formula was used to calculation the percentage of cell viability in comparison to control groups, including both un-treated and treated with sulfadiazine (40 μg/mL) plus pyrimethamine (1 μg/mL): % viable cells = (drug well absorption – blank well absorption / control well absorption – blank well absorption) ×100.

#### Evaluation of Apoptosis by Flow Cytometry

Evaluation of necrosis and apoptosis in cells were undertaken using Annexin V-FITC Apoptosis Detection Kit (Bio Vision, Palo Alto, USA). In brief, in 12-well plates, 5 × 10^5^ tachyzoites and infected macrophages were cultured separately and were exposed to 0.01 and 0.1 μg/ mL concentrations of imiquimod. Cells not exposed to drug were used as the control. Tachyzoites were incubated for 3 h, while infected macrophages were incubated for 24 h. After incubation the cells were washed in cold PBS and centrifuged at 1,000 g for 5 min. The supernatant was then drained off and replaced with 500 μL binding buffer, followed by 5 μL of annexin V and 5 μL of propidium iodide (PI). Samples were kept in the dark at a temperature of 24 ± 2°C for 5 min and were analyzed by FACSCaliber flow cytometer (BD Biosciences) with FlowJo software (Ebrahimisadr et al., 2018).

### *In vivo* Assay

#### Animal Design

Sixty BALB/C mice (female, 6–8 weeks old) were purchased from the Royan Institute (Tehran, Iran) and housed in a colony room at an ambient temperature of 20–25°C and relative humidity ranging from 55 ± 65% under a 12/12 h light/dark cycle and free access of food and water. All mouse experiments were carried out in accordance with approved protocols by the Tarbiat Modares University of Medical Sciences Ethical Laws Committee's Institutional Animal Care and Use Committee. The mice were distributed into six groups (ten animals in each cage). Mice in 5 of the groups were injected intraperitoneally with 1 × 10^4^ *T. gondii* tachyzoites, with the remaining group uninfected as a healthy control. Four hours later all groups were treated as follows: One group before challenge was treated with imiquimod (1 mg/kg, once a week, intraperitoneal injection) for 21 days and the other group received imiquimod at the same dose (1 mg/kg, once a week, intraperitoneal injection) after infection. The third group received sulfadiazine (40 mg/kg/day, orally) plus pyrimethamine (1 mg/kg/day, orally) (SDZ+PYR) and fourth group received sulfadiazine (10 mg/kg/day, orally) plus pyrimethamine (0.5 mg/kg/day, orally) in combination with imiquimod (0.5 mg/kg, once a week, intraperitoneal injection) (SDZ+PYR+IQ). The two remaining groups were healthy and infection controls and only received PBS (intraperitoneal injection) over the study period.

#### Survival Assay

To evaluate the efficacy of imiquimod against *T. gondii* infection five mice per group were randomly selected after treatment as mentioned above. Survival was checked daily and the mortality rate was documented for each group until all mice had died.

#### Quantitative Real Time PCR (qPCR) for Evaluation of Parasite Load in Spleen Tissues

Three days after treatment five mice in each group were sacrificed and their spleens were removed and immersed in 5 ml of cold PBS (pH 7.4). Subsequently, a piece of spleen tissue from each of the study groups was cut, weighed and placed in to a 1.5 mm DNase/RNase-free tubes and then homogenized with a tissue grinder. The rest of spleens were kept for cytokine assessment. The parasite numbers in spleen tissues of the mice were measured by quantitative real time PCR (qPCR) as above.

#### Cytokine Measurements

Lymphocytes were prepared by crushing spleen tissues and culturing (5 × 10^6^ cells/ml) in 24-well plates in the RPMI1640 medium (Gibco, Germany) supplemented with 10% FCS (Gibco-BRL, France), penicillin (100 unit ml−1), and streptomycin (100 μgml−1) (Sigma, Germany) under sterile conditions. For preparing *Toxoplasma* Lyzate Antigen (TLA), 2 × 10^9^ tachyzoites of *T. gondii* RH strainwere passed through 3 μm pore size filter membranes and then centrifuged at 4°C, 3 times for 15 min to separate the supernatant. Subsequently, 1 ml of protease inhibitor (phenylmethylsulphonyl fluoride) was added to the supernatant and then *T. gondii* tachyzoites were lysed through 5 freeze–thaw cycles alternately in liquid nitrogen and a water bath at 37°C. The protein concentration of TLA was obtained by Bradford method. In order to stimulate the lymphocyte cells, TLA (50 μg/ml) was added to each well followed by incubation at 37°C under 5% CO2 for 3 days. After incubation, the supernatant was harvested and stored frozen at −70°C until used for serological tests. Levels of both IL4 and IFN-γ in culture supernatants were assessed using an ELISA kit (Mabtag, Germany) following the manufacturer's recommended procedure (Zaki et al., 2020).

#### Date Analysis and Statistics

Statistical analyses were accomplished using IBM SPSS Statistics Version 21 and graphical presentation of data are performed using Graph Pad Prism version 8.0.1 and Microsoft Office Excel 2013. Data were presented as the mean, standard deviation (SD), and 95% confidence interval (CI). In order to determine statistical significance, analysis of variance (One-Way-ANOVA) was employed. The Kaplan-Meier estimate was done for evaluation of survival rate. In this work parametric tests such as unpaired samples *t*-test were used for the comparison of the results between the treatment and control groups. Differences were presumed significant compared to control at 5% (*p* < 0.05).

## Results

### Tachyzoite Assay by Microscopic Observation

**Figure 1:**
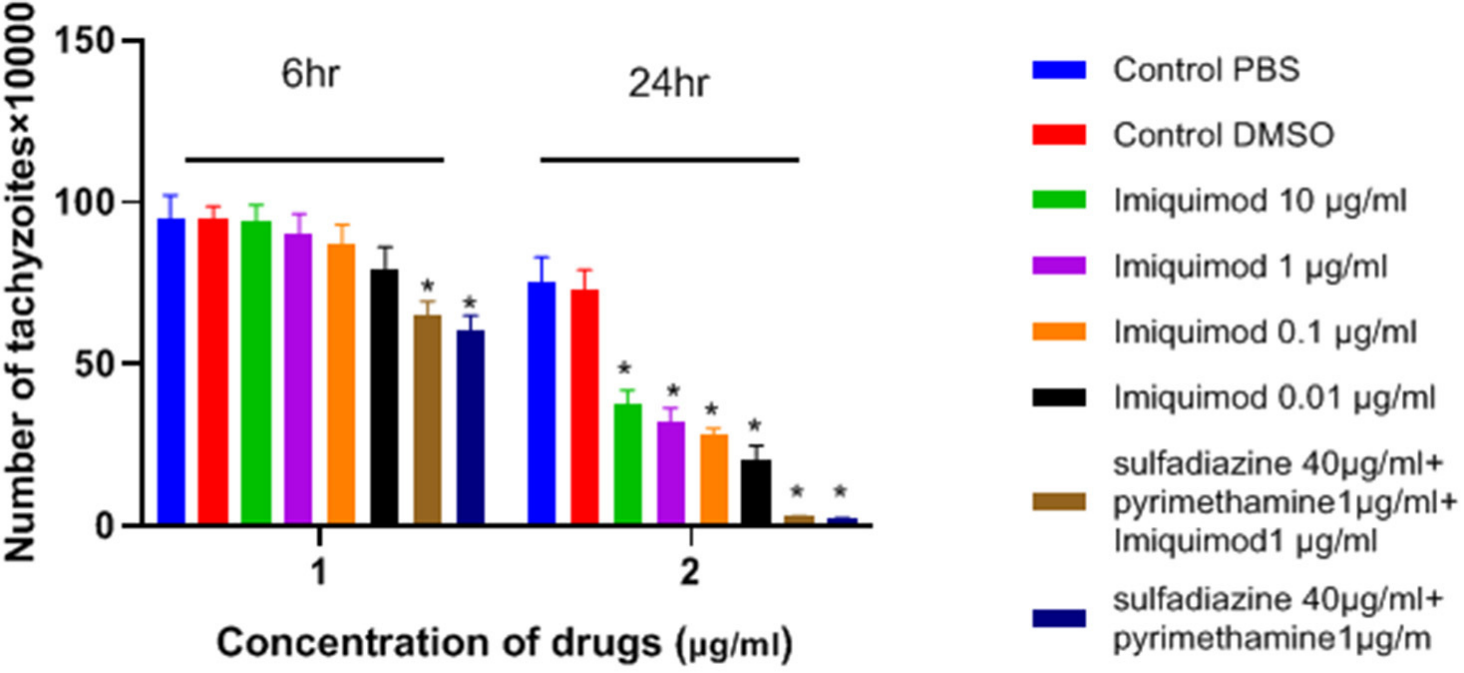
Mean and standard deviation of the number of *T. gondii* tachyzoites exposed different concentrations of imiquimod for 6 and 24 h compared to control groups (sulfadiazine plus pyrimethamine and untreated). There is statistical difference between control groups and treated groups (^*^P < 0.05 compared to controls).

As shown in Figure 1, the number of tachyzoites declined significantly in the presence of all concentrations of imiquimod compared to those in control group after 24 h (*p* < 0.05). The maximum effect was observed after 24 h with imiquimod 0.01 μg/ml, while the least effect was seen with imiquimod 10 μg/ml. Toxic effects of imiquimod on tachyzoites were found to be both dose- and time dependent. Additionally, it was shown that the anti-*Toxoplasma* effects of imiquimod in combination with sulfadiazine plus pyrimethamine (IQ+SDZ+PYR) were similar to positive control group (SDZ+PYR).

### Uninfected Macrophage Viability Test

**Figure 2:**
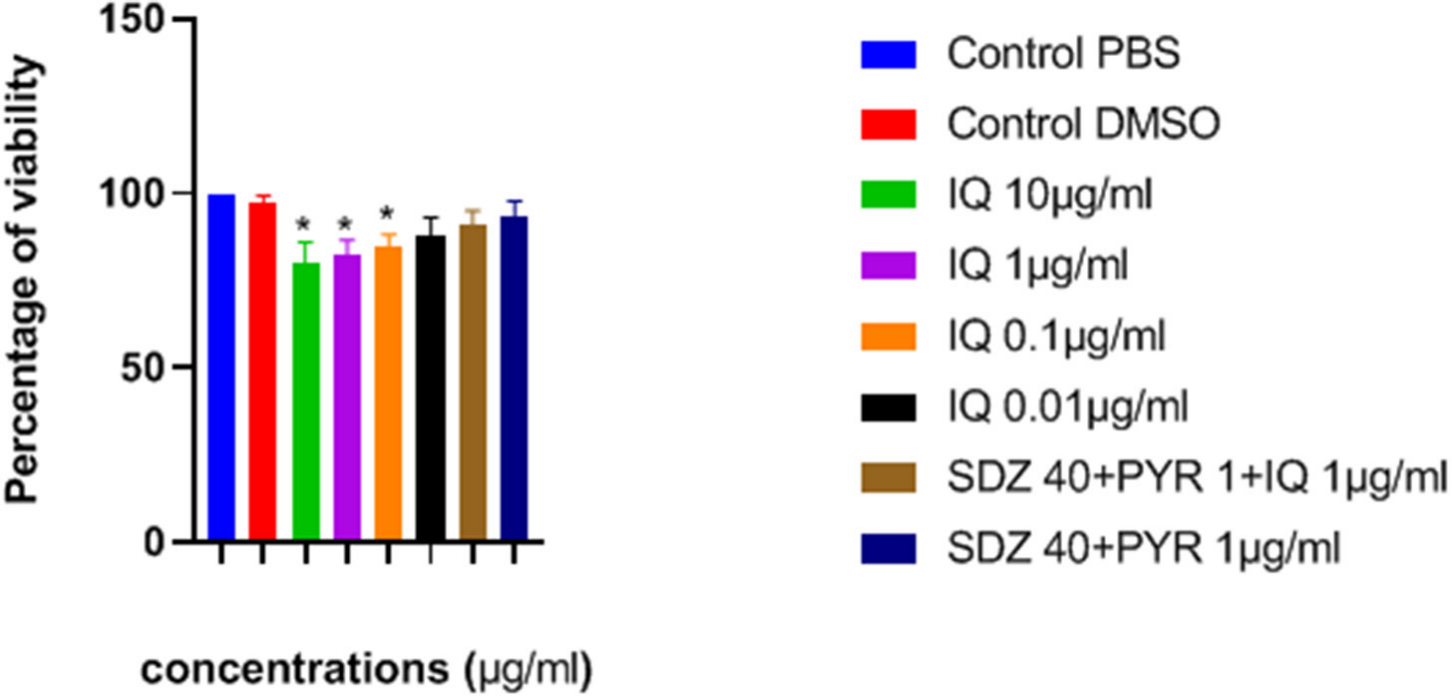
Mean and SD of percentage of macrophage viability exposed to different concentrations of drugs after 24 h. There is statistical difference with control group (*P < 0.05). IQ, Imiquimod; SDZ, Sulfadiazine; PYR, pyrimethamine.

The effects of imiquimod on uninfected macrophages were investigated by measurement of optical density (OD) following MTT assay. Lower concentrations of drug (e.g., 0.01 and 0.1 μg/ mL) were associated with greater viability after 24 h (Figure 2). Additionally, viability of both sulfadiazine 40 μg/mL plus pyrimethamine 1 μg/mL (SDZ+PYR) alone and in combination with imiquimod 1 μg/mL (SDZ+PYR+IQ) was good (93 and 90.9%, respectively).

### Flow Cytometry Analysis

**Figure 3:**
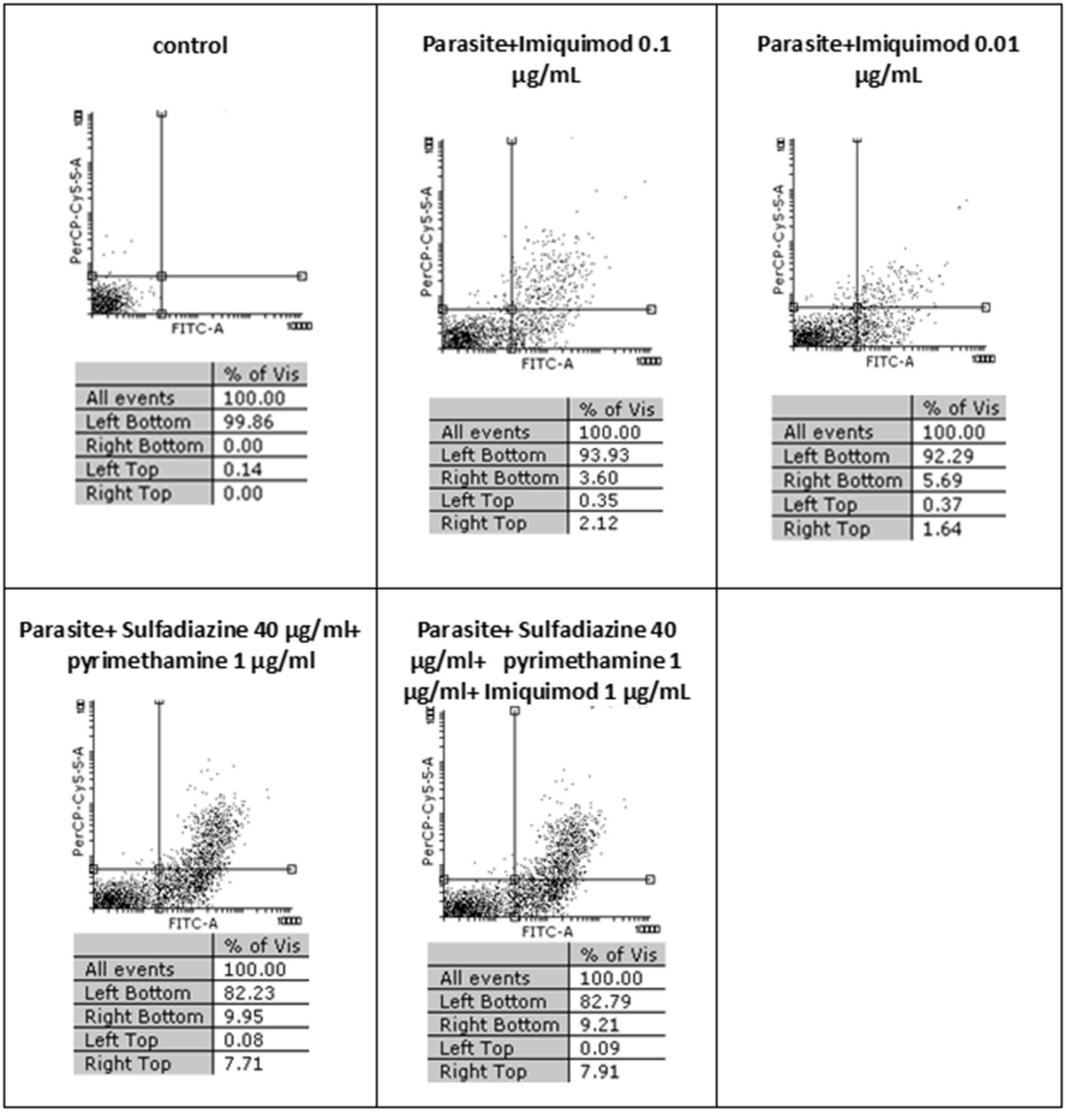
The effect of different concentrations of imiquimod on *T. gondii* tachyzoites viability and comparing them with the control group (untreated) after 3 h. Regions of quadrat shows: necrosis cells (propidium iodide positive) in left top, late apoptosis in right top, apoptotic cells (annexin positive) in right bottom and live cells in left bottom.

**Figure 4:**
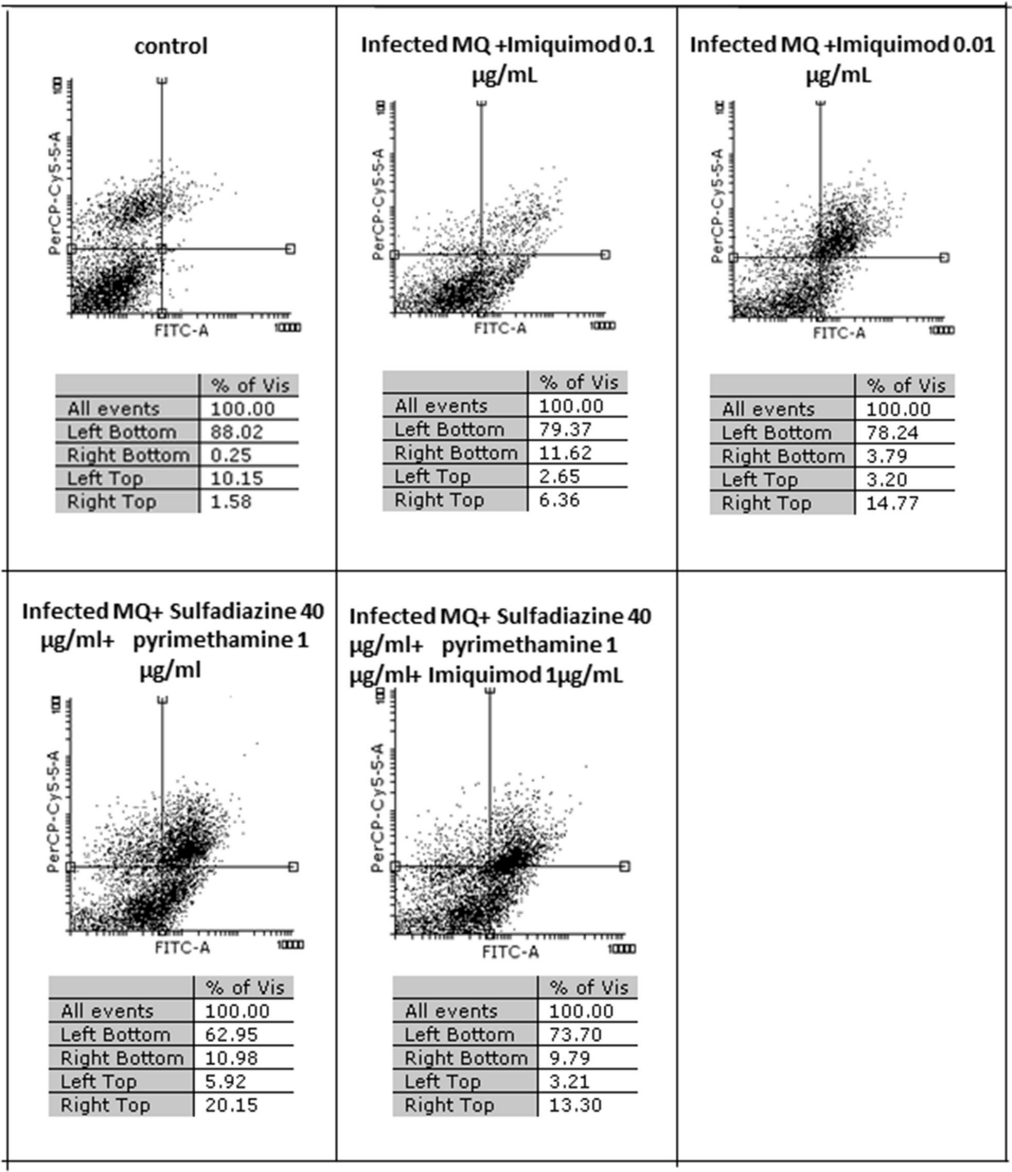
The effect with 2 concentrations (1 and 0.01 μg/ml) of imiquimod and sulfadiazine/pyrimethamine with and without imiquimod on infected macrophage viability compared to untreated control after 24 h. Regions of quadrat shows: necrosis cells (propidium iodide positive) in left top, late apoptosis in right top, apoptotic cells (annexin positive) in right bottom and live cells in left bottom.

Flow cytometric analysis showed that apoptosis after 3 h exposure of tachyzoites to 0.01 and 0.1 μg/mL imiquimod (7.3 and 5.7%, respectively) did not differ significantly from the control group (*P* < 0.05) (Figure 3). Sulfadiazine plus pyrimethamine (SDZ+PYR) alone and in combination with imiquimod (SDZ+PYR+IQ) induced more apoptosis but was not significantly different from control. After 24 h, treatment of infected macrophages with 0.01 or 0.1 μg/mL imiquimod induced apoptosis in 18.66 and 18.00% respectively, in comparison to 1.8% in control (untreated) macrophages. Most apoptosis occurred in sulfadiazine plus pyrimethamine (SDZ+PYR) alone and in combination with imiquimod (SDZ+PYR+IQ) groups (Figure 4).

### Measurement of Parasite Load of Macrophage Cells Using Quantitative Real Time PCR (qPCR)

**Figure 5:**
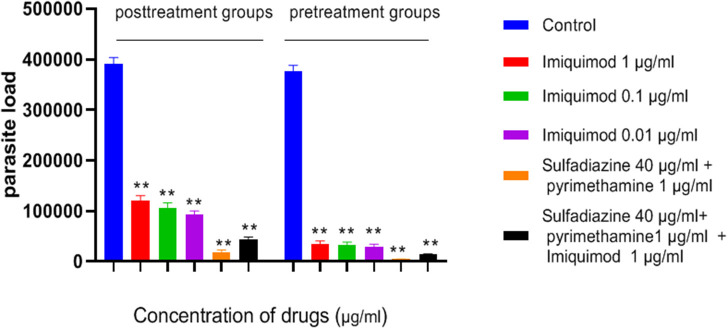
Parasite load of macrophage cells before and after treated with drugs. Values are mean ± standard deviation of two independent experiments. **P < 0.01 compared with untreated group.

Table 1 and Figure 5 show the results of qPCR analysis for macrophages treated pre- and post-infection after 24 h incubation. The results showed that parasite loads in both groups treated with all concentrations of drugs were reduced in comparison with untreated macrophages (negative control). Additionally, a very large decrease was observed after 24 h in parasite load in macrophages treated with imiquimod before infection (*p* < 0.001).

**Table 1:** Cycle of Threshold (CT) and parasite load test copy/reaction according to Real Time PCR method for macrophages before and after infection with 1 × 10^4^ tachyzoite forms of *T. gondii* RH strain and control groups.

**Groups**	**Parasite load of macrophage cells before**		**Parasite load of macrophage cells after**	
	**treated with imiquimod**		**treated with imiquimod**	
	**Cycle of threshold for test**	**Parasite load test copy/reaction**	**Cycle of threshold for test**	**Parasite load test copy/reaction**
Control (healthy macrophages)	30.50	0	29.76	0
Control (infected macrophages)	11.71	376435	10.54	391075
Imiquimod 1 μg/ml	18.21	33684	16.10	119623
Imiquimod 0.1 μg/ml	18.27	32500	16.18	106544
Imiquimod 0.01 μg/ml	18.51	29363	16.53	92671
Sulfadiazine 40 μg/ml + pyrimethamine 1 μg/ml	21.70	4222	20.80	18465
Sulfadiazine 40 μg/ml + pyrimethamine1 μg/ml + Imiquimod 1 μg/ml	22.20	10784	17.98	39608

### Quantification of Parasite Load of Spleen Tissues Using Quantitative Real Time PCR (qPCR)

The spleen parasite burden for all groups was evaluated by comparison with the standard curve and quantitative real-time PCR (qPCR) analysis. Animals receiving imiquimod before being challenged with tachyzoites (pre-treatment group) showed lower parasite loads than those receiving drug after infection (post-treatment groups). Reduced parasite load was found in all treated mice in both groups (pre-treatment and post-treatment groups), and therefore statistically significant difference was seen between drug and control groups (*p* < 0.001). The parasite load decreased significantly in groups treated with sulfadiazine plus pyrimethamine (SDZ+PYR) alone and in combination with imiquimod (SDZ+PYR+IQ) compared with the groups receiving PBS (*p* < 0.001) (Table 2).

**Table 2:** Cycle of Threshold (CT) and parasite load of Spleen Tissues according to Quantitative real time PCR (qPCR) method for all treated and control groups.

**Number**	**Sample type**	**Cycle of threshold**	**Parasite load test**
**test**		**for test**	**copy/reaction**
1	Control (healthy mice)	30.53	0.04
2	Control (infected mice)	12.93	132545
3	Imiquimod before challenge	25.81	192
4	Imiquimod after challenge	17.21	14440
5	Sulfadiazine+pyrimethamine	28.28	0.22
6	Sulfadiazine+pyrimethamine + Imiquimod	28.15	0.51

### Determination of Cytokine Secretion Levels

**Figure 6:**
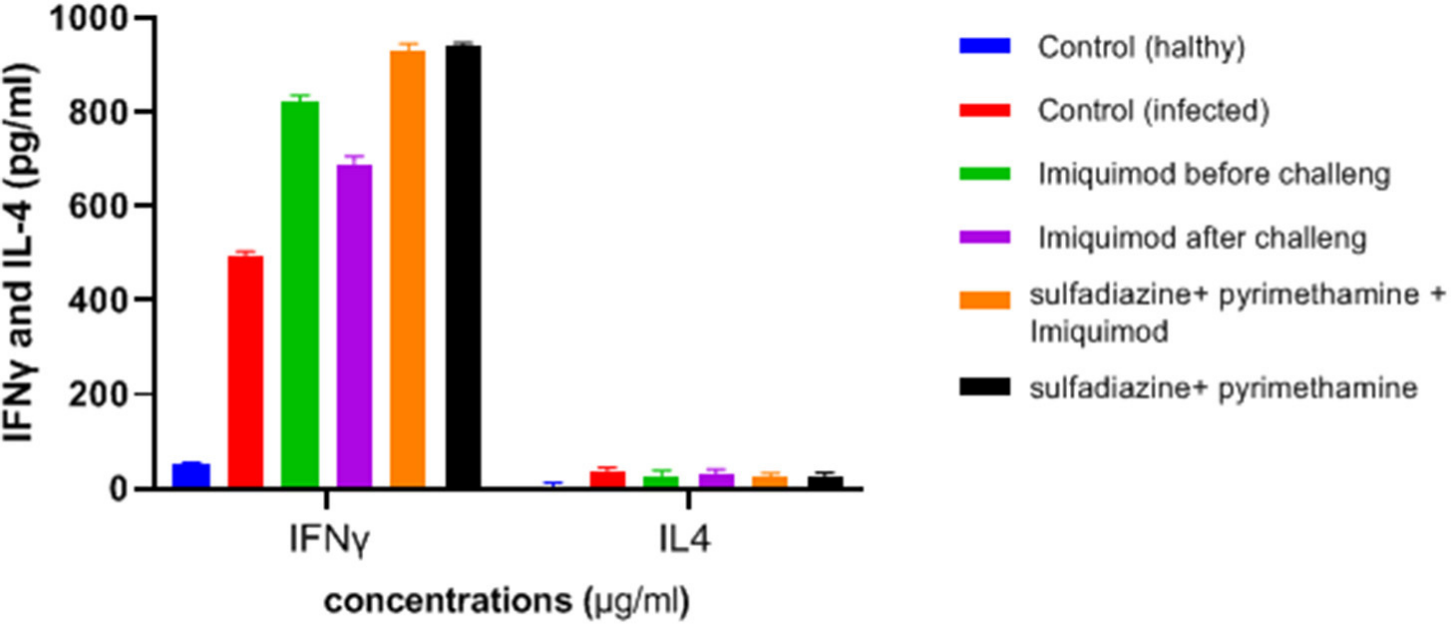
Levels of IFNγ and IL-4 (pg/ml) cytokines in spleen lymphocyte culture in test and control groups after 72 h stimulation with *Toxoplasma* Lyzate Antigen. SDZ, Sulfadiazine (40 mg/kg/day); PYR, pyrimethamine (1 mg/kg/day); IQ, Imiquimod (1 mg/kg, once a week).

ELISA results showed an increasing trend in the IFNγ cytokine levels among all treated groups in comparison with the untreated groups (*P* < 0.05) (Figure 6). It was found that the mean IFN-γ level in animals treated with imiquimod before challenge with parasite was significantly higher than the values obtained for other groups (*P* < 0.05). Also, the highest mean IFN-γ levels after 3 days of exposure was related to the mice treated with sulfadiazine plus pyrimethamine (SDZ+PYR) alone and in combination with imiquimod (SDZ+PYR+IQ) in comparison with other groups (*P* < 0.05). A marked decrease in IL4 production was detected in treated animals compared to control mice.

### Survival Rate Measurement

**Figure 7:**
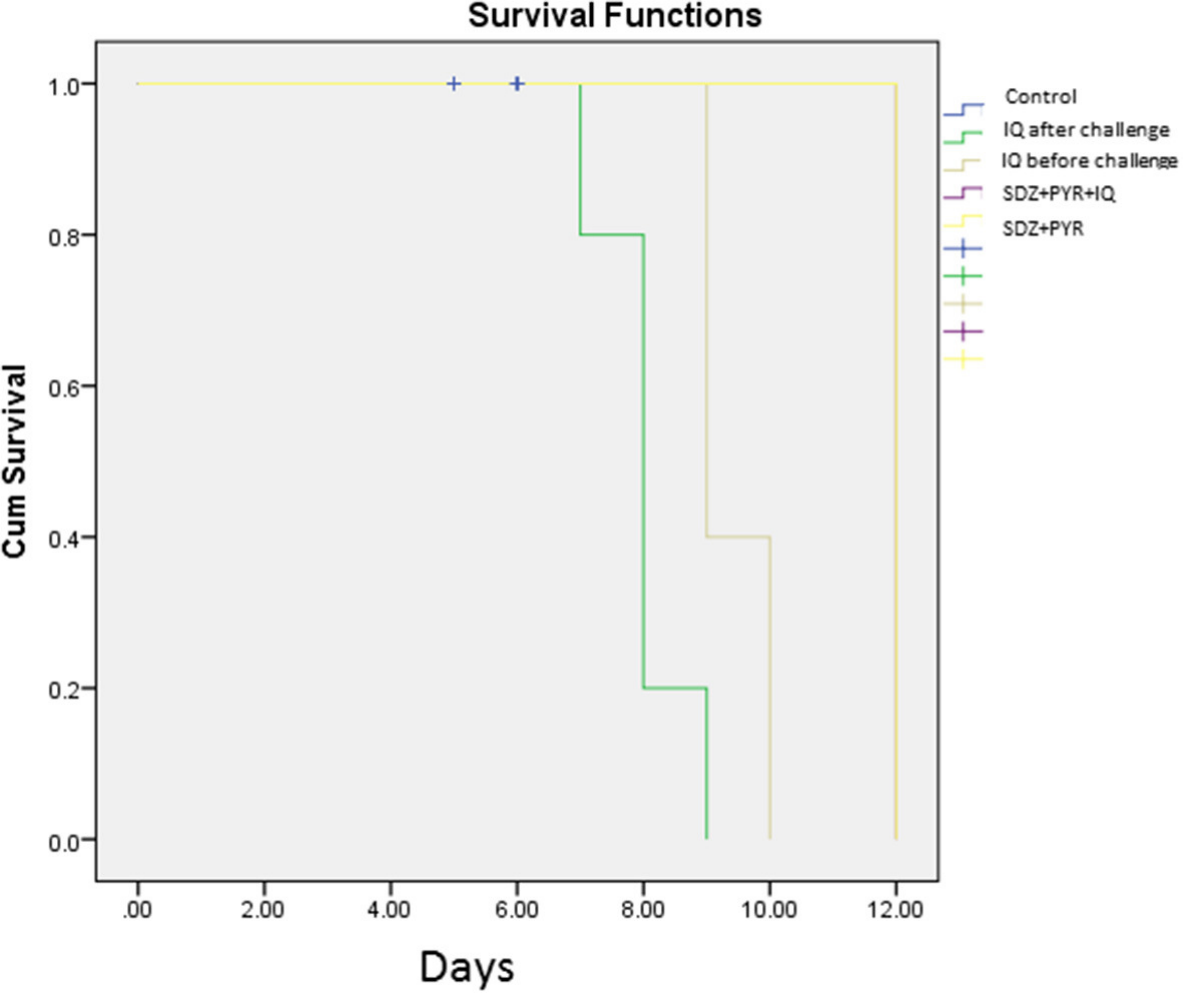
Survival rates of treated and control BALB/c mice after and before challenge with 1 × 10^4^ tachyzoite forms of *T. gondii* RH strain (5 mice per group).

Evaluation of survival rate in mice (5 animals/group) was undertaken over 4–12 days after the infection. Death in the untreated mice occurred from day 5 after infection and all had died by day 6 of the study. Mice treated with imiquimod before infection showed prolonged survival time compared with post-treated and control groups (*P* < 0.05). Moreover, all mice were alive following post infection treatment with sulfadiazine plus pyrimethamine (SDZ+PYR) alone and in combination with imiquimod (SDZ+PYR+IQ) (Figure 7).

## Discussion

Severe and fatal complications of toxoplasmosis remains a problem that lacks a global solution. Current treatment for *T. gondii* infection fails to achieve therapeutic goals owing to poor tolerability, limited efficacy and cost (Maubon et al., 2010; Pinzan et al., 2015). As there are no effective therapeutic strategies available to improve early treatment *T. gondii* infection, the development of new anti-*Toxoplasma* drugs should have high priority to tackle toxoplasmosis and reduce infection.

Imidazoquinoline compounds such as imiquimod work as immune response modifiers with antiproliferative, antiparasite, antifungal, and antiviral activities that have been examined in several previous studies (van der Fits et al., 2009; de Sousa et al., 2014; Tio et al., 2018; Jabari et al., 2019). The cytotoxic effect of imiquimod against various cancers has been investigated, and it was shown that it induces apoptosis as well as increasing levels of the opioid growth factor receptor (OGFr) (Wybran and Plotnikoff, 1991; Zagon et al., 2008). It is also well established that imiquimod contributes to both innate and cell-mediated immunity through activation of toll-like receptor 7 (TLR7) macrophages, monocytes and dendritic cells (Hemmi et al., 2002; Jabari et al., 2019). Consequently, stimulation of immune cells by imiquimod leads to the production of cytokines such as interferon-α (IFN-α), interleukin-6 (IL-6), tumor necrosis factor-α (TNF-α), essential to elicit immune responses (Buates and Matlashewski, 1999; Khamesipour, 2014). Although a number of studies have demonstrated the anti-parasitic properties of imiquimod (Mehravaran et al., 2020), there have been no investigations of its effect on *Toxoplasma* infections. In this regard, imiquimod alone or in combination with other compounds reduced proliferation of promastigotes and amastigotes of *L. major in vitro* and *in-vivo*. It was concluded that this drug could be useful to treatment of leishmaniasis by modulating the immune reaction preferentially toward a Th1-predominant state (Buates and Matlashewski, 1999, 2001; Mehravaran et al., 2020).

Jabari et al. (2019) investigated the *in-vitro* effect of imiquimod against *L. major* promastigotes and amastigotes. Imiquimod either singly or in combination had a pronounced effect in reduction and prevention of macrophage infection with amastigotes (Jabari et al., 2019). Ebrahimisadr et al. (2019) assessed antileishmanial effects of imiquimod on *L. major* using Real-Time PCR and reported a significant reduction in the mean lesion size in BALB/c mice. Additionally, their results showed a significant increase in expression of TLR2 and TLR4 genes in lesion RNA extracted from infected mice (Ebrahimisadr et al., 2018). The anti-leishmanial activity of monophosphoryl lipid A and imiquimod adjuvants and soluble *Leishmania* antigen in a nano-liposome carrier was evaluated by Emami et al. (2018), who reported that this combination could be an appropriate delivery system to induce the cellular immune pathway against *L. major* infection in BALB/c mice (Emami et al., 2018).

In the present study, the effectiveness of different concentrations of the imiquimod against *T. gondii* tachyzoites was investigated and showed imiquimod reduced the number of tachyzoites in a concentration- and time-dependent manner. Notably, the maximum tachyzoites death occurred with a imiquimod concentration of 0.01 μg/ml, after 24 h. Considerable toxicity was observed for *T. gondii* tachyzoites of treated with sulfadiazine plus pyrimethamine alone or in combination with imiquimod (SDZ+PYR+IQ). Toxicity of imiquimod in uninfected macrophages was dose-dependent; The MTT assay showed that as concentrations of imiquimod were decreased, viability of uninfected macrophages increased. It had previously been demonstrated that imiquimod could induce apoptosis in uninfected macrophages as well as parasites (Ebrahimisadr et al., 2018; Jabari et al., 2019). Induction of programed cell death occurred in both tachyzoites and in infected macrophages when treated with imiquimod, although these apoptotic effects were not remarkable. Also, necrosis was low in infected macrophages treated with imiquimod compared to healthy macrophages without treatment. Therefore, this drug may have positive effects against toxoplasmosis infection through augmentation of the immune response as well as expression of cell level receptors. Under *in vitro* conditions our experiment demonstrated that adjuvant and/or preventive effects of imiquimod can decrease the burden of parasites in macrophages. In this regard, when treatment of macrophages with imiquimod was done before infection, a remarked reduction in parasite burden was observed. Interestingly, the lowest parasite burden occurred with a concentration of 0.01 μg /mL imiquimod.

Macrophages as antigen-presenting cells are the primary site of action for imiquimod and have a pivotal role to recognize *T. gondii*-infected cells (Buates and Matlashewski, 2001; Innes et al., 2019). Therefore, it can be concluded that imiquimod has the ability to limit toxoplasmosis by generating a powerful immune response following induction of macrophages. In the murine model, the parasitic burden and mortality rate in BALB/c mice that received imiquimod before parasitic challenge was lower than those received drug after parasitic challenge. Overall, the lowest parasite burden and mortality rate was seen in mice treated with sulfadiazine plus pyrimethamine in combination with imiquimod (SDZ+PYR+IQ).

Both humoral and cellular immune responses must be stimulated in order to generate protection against *T. gondii* infection (Foroutan et al., 2019). In the mouse model, production of from immune system cells is required for both innate and adaptive immune responses. IFN-γ plays a fundamental role in limiting toxoplasmosis by enhancing the defensive function of macrophages and inducing a strong Th1 type immune response (Suzuki et al., 1988; Zhang et al., 2015; Innes et al., 2019). The present study indicated that a TH1-type cellular immune response was induced in all treated mice with production of IFN-γ and prolonged survival time compared to the untreated control group. In addition, the imiquimod treated mice before infection also displayed greater levels of IFN-γ compared to the control group. However, levels of IL-4 production were low among treated mice.

These results demonstrate a significant anti-*Toxoplasma* activity for imiquimod against *T. gondii* tachyzoites and infected macrophages. Imiquimod in the pretreated group had greater anti-*Toxoplasma* effects than imiquimod in post-treated group *in vitro* and *in vivo*. The *in vivo* experiments showed that it can trigger a strong immune response by enhancing IFN-γ level and survival time and by decreasing the parasite load in BALB/c mice. Therefore, it can be inferred that imiquimod could be used as an adjunctive treatment for the treatment of disease due to *T. gondii*. Imiquimod should therefore be considered for further study either alone or along with other anti-*Toxoplasma* products in future studies.

## Data Availability Statement

The original contributions presented in the study are included in the article/supplementary material, further inquiries can be directed to the corresponding author/s.

## Ethics Statement

The animal study was reviewed and approved by Ethical Committee of Tarbiat Modares University approved this study.

## Author Contributions

FG was supervisor of this research, conceived the study, and designed the study protocol. LZ performed the experiments, analyzed the data, and drafted the manuscript. JH the advisor of project, designed the study protocol and critically revised the manuscript. ZS and JS were advisors of the research. All authors read and approved the final version of the manuscript.

## Conflict of Interest

The authors declare that the research was conducted in the absence of any commercial or financial relationships that could be construed as a potential conflict of interest.
